# Crystal structure of 3-de­oxy-3-nitro­methyl-1,2;5,6-di-*O*-iso­propyl­idene-α-d-allo­furan­ose

**DOI:** 10.1107/S2056989016001845

**Published:** 2016-02-10

**Authors:** Jevgeņija Lugiņina, Vitālijs Rjabovs, Dmitrijs Stepanovs

**Affiliations:** a Institute of Technology of Organic Chemistry, Faculty of Materials Science and, Applied Chemistry, Riga Technical University, P. Valdena 3/7, Riga, LV-1048, Latvia; b Latvian Institute of Organic Synthesis, Str. Aizkraukles 21, Riga, LV 1006, Latvia

**Keywords:** crystal structure, nitro carbohydrate, nitro sugar, *C*(3)-nitro­methyl allose, allo­furan­ose, C—H⋯O hydrogen bonding

## Abstract

The title compound, C_13_H_21_NO_7_ {systematic name: (3a*R*,5*S*,6*R*,6a*R*)-5-[(*R*)-2,2-dimethyl-1,3-dioxolan-4-yl]-2,2-dimethyl-6-(nitro­meth­yl)tetra­hydro­furo[2,3-*d*][1,3]dioxole}, consists of a substituted 2,2-di­methyl­tetra­hydro­furo[2,3-*d*][1,3]dioxolane skeleton. The furan­ose ring *A* adopts a *^o^T*_4_ conformation. The fused dioxolane ring *B* and the substituent dioxolane ring *C* also have twisted conformations. There are no strong hydrogen bonds in the crystal structure: only weak C—H⋯O contacts are present, which link the mol­ecules to form a three-dimensional structure.

## Abstract 2

The title compound, a nitro carbohydrate, consists of a substituted 2,2-di­methyl­tetra­hydro­furo[2,3-*d*][1,3]dioxolane skeleton. The furan­ose ring adopts a *^o^T*_4_ conformation.

## Chemical context   

The title compound **1**, has been used for the syntheses of isoxazoles (Lugiņina *et al.*, 2013 ▸) and carbohydrate-based amines and amino acids (Rjabovs *et al.*, 2015*a* ▸). Carbohydrates with amino groups are valuable synthetic precursors and are easily converted to spiro­cyclic carbohydrate derivatives (Turks *et al.*, 2013 ▸), imino sugars (Filichev & Pedersen, 2001 ▸), nucleic acid mimetics (Rozners *et al.*, 2003 ▸), and azido sugars (Mackeviča *et al.*, 2014 ▸; Rjabova *et al.*, 2012 ▸). The latter are widely used for the syntheses of triazoles (Uzuleņa *et al.*, 2015 ▸; Grigorjeva *et al.*, 2015 ▸) and THF-amino acids (sugar amino acids) (Rjabovs & Turks, 2013 ▸).
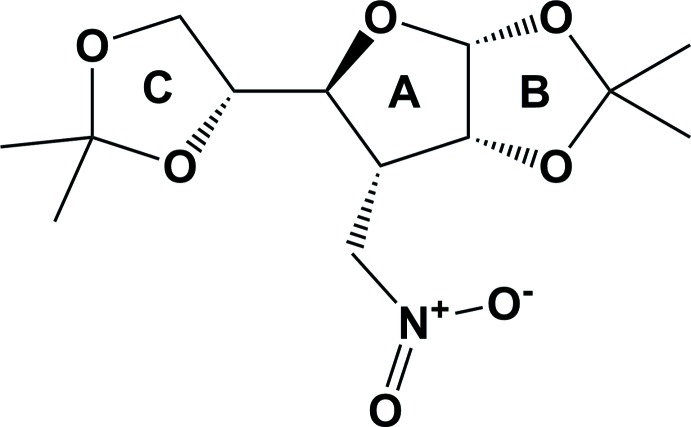


## Structural commentary   

**Figure 1:**
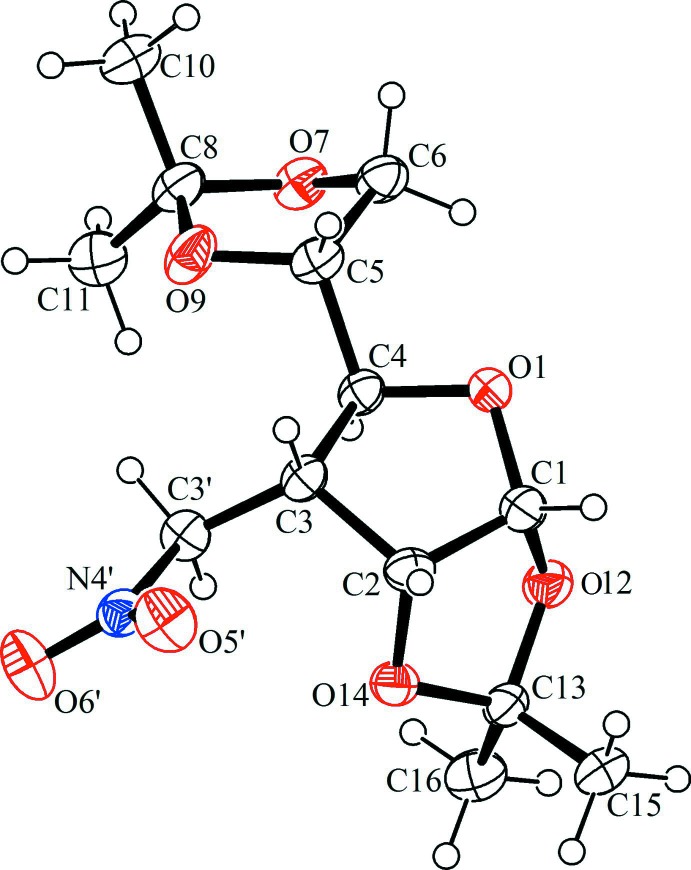
The mol­ecular structure of the title compound **1**, showing the atom labelling. Displacement ellipsoids are drawn at the 50% probability level.

The title compound **1**, consists of a tetra­hydro­furan core (ring *A*) fused with a dioxolane ring (*B*) and substituted with a dioxolane (ring *C*) and a nitro­methyl group (Fig. 1 ▸). The conformational analysis of the furan­ose ring (*A*) based on the inter­nal dihedral angles of the ring shows that its pseudo­rotational phase angle *P* = 70° (Altona & Sundaralingam, 1972 ▸; Taha *et al.*, 2013 ▸). Thus, this ring adopts a conformation close to ^o^ *T*_4_, where O1 and C4 deviate by 0.214 (2) and −0.340 (3) Å, respectively, from the plane through atoms C1/C2/C3. Such a conformation of the furan­ose ring is rather unusual for 3-*C*-monosubstituted 3-de­oxy-1,2-*O*-iso­propyl­idene-α-d-allo­furan­oses. For example, previously reported structures (Rjabovs *et al.*, 2014 ▸, 2015*a* ▸,*b* ▸) had conformations between ^3^ *E* and ^3^ *T*_4_. The fused dioxolane ring *B* also adopts a twisted conformation on bond C13—O12; these atoms deviate by −0.324 (4) and 0.224 (3) Å, respectively, from plane C1/C2/O14. The dihedral angle subtended by the mean planes of rings *A* and *B* is 63.7 (2)°. The five-membered ring of the 2,2-dimethyl-1,3-dioxolan-4-yl group, ring *C*, also adopts a twisted conformation, on bond C6—O7; these atoms deviate by 0.143 (4) and −0.381 (2) Å, respectively from plane C5/O9/C8.

## Supra­molecular features   

**Figure 2:**
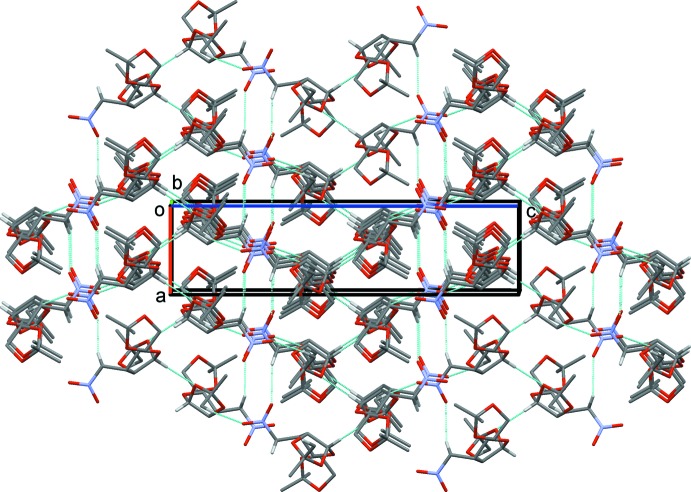
A view along the *b* axis of the crystal packing of the title compound **1**. Hydrogen bonds are shown as dashed lines (see Table 1 ▸) and H atoms not involved in these inter­actions have been omitted for clarity.

In the crystal, mol­ecules are linked *via* C—H⋯O hydrogen bonds, forming chains along [100]. The chains are linked *via* further C—H⋯O hydrogen bonds, forming a three-dimensional structure (Table 1 ▸ and Fig. 2 ▸).

## Database survey   

**Figure 3:**
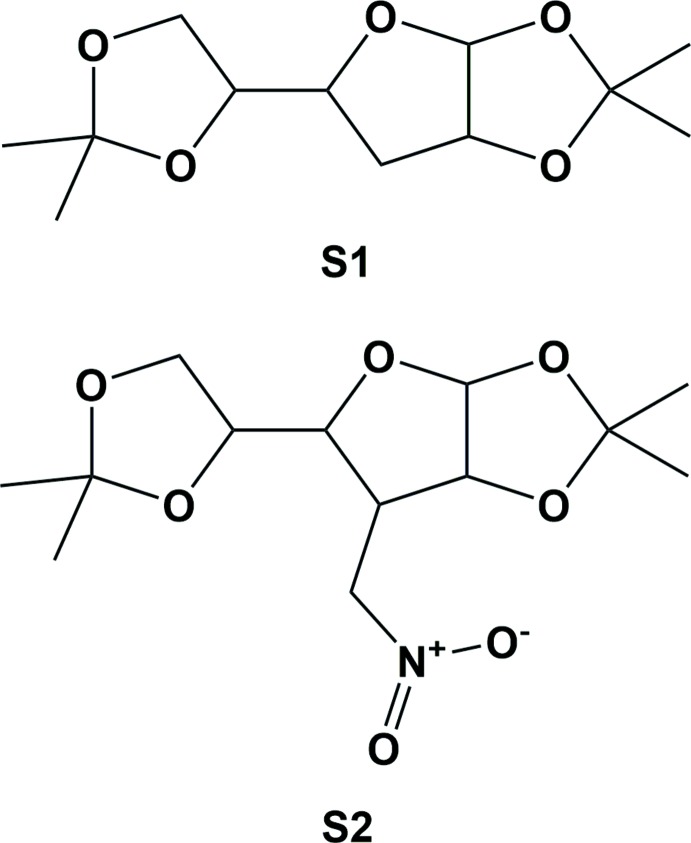
Substructures used for the database survey.

A search of the Cambridge Structural Database (Version 5.37; Groom & Allen, 2014 ▸) for substructure S1 (Fig. 3 ▸) gave 137 hits, while a search for substructure S2 (Fig. 3 ▸) gave only five hits. Amongst the latter compounds, four concern the structures with a hydroxyl and a nitro­methyl group attached to atom C3 (BOGFOU: Turks *et al.*, 2014 ▸; BOGFUA: Turks *et al.*, 2014 ▸; CIDVO: Turks *et al.*, 2013 ▸; USODEM: Zhang *et al.*, 2011 ▸). They are diastereomers crystallizing in space groups *P*2_1_2_1_2_1_, *P*3_2_, *C*2 and *P*6_1_, respectively. In the fifth compound (KATWIN; Lugiņina *et al.*, 2012 ▸), the extra substituent at atom C3 is a methylthio group; it crystallizes in space group *C*2.

## Synthesis and crystallization   

**Figure 4:**
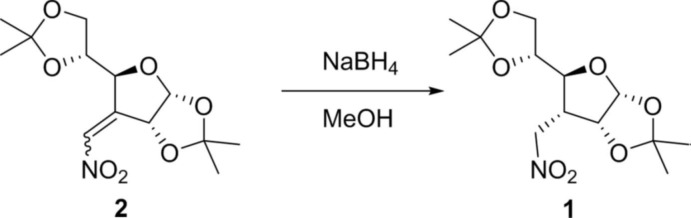
Synthesis of the title compound.

The title compound **1**, was synthesized by reduction of the nitro olefin **2** (Albrecht & Moffatt, 1970 ▸; Filichev *et al.*, 2001 ▸; Lugiņina *et al.*, 2012 ▸) with sodium borohydride in methanol solution, as illustrated in Fig. 4 ▸. NaBH_4_ (6.2 g, 163.9 mmol, 5.5 eq.) was added portion wise to a solution of **2** (9.1 g, 30.0 mmol, 1.0 eq.) in MeOH (200 ml) over 30 min at 273 K. After completion (monitored by TLC) the reaction mixture was acidified using 10% aqueous solution of AcOH to pH 6–7 and then evaporated to dryness. The residue was dissolved in EtOAc (90 ml), washed with brine (3 × 10 ml), dried over NaSO_4_, and evaporated. The product **1** was purified by column chromatography on silica gel (Hexanes/EtOAc 3:1 → 2:1) giving a white crystalline solid (yield: 6.5 g, 72%; m.p. 355–356 K). *R_f_* = 0.9 (hexa­nes/EtOAc 1:1). ^1^H NMR (CDCl_3_, 300 MHz): δ 5.84 (*d*, *J* = 3.7 Hz, 1H), 4.88–4.82 (*m*, 21H), 4.68 (*dd*, AB syst., *J* = 14.9 Hz, *J* = 10.4 Hz, 1H), 4.14 (*dd*, *J* = 8.0 Hz, *J* = 5.5 Hz, 1H), 4.02–3.92 (*m*, 2H), 3.65 (*dd*, *J* = 9.9 Hz, *J* = 8.4 Hz, 1H), 2.74 (*tt*, *J* = 10.1 Hz, *J* = 4.4 Hz, 1H), 1.52 (*s*, 3H), 1.40, (*s*, 3H), 1.33 (*s*, 3H), 1.32 (*s*, 3H). ^13^C-NMR (75 MHz, CDCl_3_): δ 112.6, 110.1, 105.4, 80.5, 79.0, 77.8, 70.8, 68.2, 46.6, 26.8, 26.7, 26.4, 25.1. X-ray quality single crystals were obtained by slow evaporation of a di­chloro­methane solution at ambient temperature.

## Refinement   

Crystal data, data collection and structure refinement details are summarized in Table 2 ▸. The H atoms were included in calculated positions and refined as riding atoms: C—H = 0.96–0.98 Å with *U*_iso_(H) = 1.5*U*_eq_(C-meth­yl) and 1.2*U*_eq_(C) for other H atoms. The absolute configuration is based on that of the starting material.

## Supplementary Material

Crystal structure: contains datablock(s) I. DOI: 10.1107/S2056989016001845/su5270sup1.cif

Structure factors: contains datablock(s) I. DOI: 10.1107/S2056989016001845/su5270Isup2.hkl

CCDC reference: 1451051

Additional supporting information:  crystallographic information; 3D view; checkCIF report

## Figures and Tables

**Table 1:** Hydrogen-bond geometry (Å, °)

*D*—H⋯*A*	*D*—H	H⋯*A*	*D*⋯*A*	*D*—H⋯*A*
C3′—H3′2⋯O5′^i^	0.97	2.53	3.355 (4)	143
C1—H1⋯O12^ii^	0.98	2.41	3.386 (5)	178
C15—H15*A*⋯O6′^iii^	0.96	2.48	3.433 (5)	174

Symmetry codes: (i) 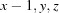; (ii) 
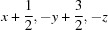
; (iii) 
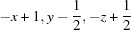
.

**Table 2:** Experimental details

Crystal data	
Chemical formula	C_13_H_21_NO_7_
*M*_r_	303.31
Crystal system, space group	Orthorhombic, *P*2_1_2_1_2_1_
Temperature (K)	173
*a*, *b*, *c* (Å)	5.5044 (2), 12.6144 (4), 21.6348 (9)
*V* (Å^3^)	1502.21 (10)
*Z*	4
Radiation type	Mo *K*α
μ (mm^−1^)	0.11
Crystal size (mm)	0.26 × 0.08 × 0.06

Data collection	
Diffractometer	Nonius KappaCCD
No. of measured, independent and observed [*I* > 2σ(*I*)] reflections	4225, 4225, 2316
(sin θ/λ)_max_ (Å^−1^)	0.705

Refinement	
*R*[*F* ^2^ > 2σ(*F* ^2^)], *wR*(*F* ^2^), *S*	0.065, 0.127, 1.01
No. of reflections	4225
No. of parameters	194
H-atom treatment	H-atom parameters constrained
Δρ_max_, Δρ_min_ (e Å^−3^)	0.20, −0.26

Computer programs: *KappaCCD Server Software* (Nonius, 1997 ▸), *DENZO* and *SCALEPACK* (Otwinowski & Minor, 1997 ▸), *SIR2011* (Burla *et al.*, 2012 ▸), *SHELXL2014* (Sheldrick, 2015 ▸), *ORTEP-3 for Windows* (Farrugia, 2012 ▸), *PLATON* (Spek, 2009 ▸) and *publCIF* (Westrip, 2010 ▸).

## supplementary crystallographic information

### Crystal data

**Table:**

C_13_H_21_NO_7_	*D*_x_ = 1.341 Mg m^−^^3^
*M**_r_* = 303.31	Mo *K*α radiation, λ = 0.71073 Å
Orthorhombic, *P*2_1_2_1_2_1_	Cell parameters from 26214 reflections
*a* = 5.5044 (2) Å	θ = 1.0–30.0°
*b* = 12.6144 (4) Å	µ = 0.11 mm^−^^1^
*c* = 21.6348 (9) Å	*T* = 173 K
*V* = 1502.21 (10) Å^3^	Needle, colourless
*Z* = 4	0.26 × 0.08 × 0.06 mm
*F*(000) = 648	

### Data collection

**Table:**

Nonius KappaCCD diffractometer	2316 reflections with *I* > 2σ(*I*)
Radiation source: fine-focus sealed tube	θ_max_ = 30.1°, θ_min_ = 3.2°
φ and ω scan	*h* = −7→7
4225 measured reflections	*k* = −17→17
4225 independent reflections	*l* = −30→30

### Refinement

**Table:**

Refinement on *F*^2^	0 restraints
Least-squares matrix: full	Hydrogen site location: inferred from neighbouring sites
*R*[*F*^2^ > 2σ(*F*^2^)] = 0.065	H-atom parameters constrained
*wR*(*F*^2^) = 0.127	*w* = 1/[σ^2^(*F*_o_^2^) + (0.0367*P*)^2^ + 0.3523*P*] where *P* = (*F*_o_^2^ + 2*F*_c_^2^)/3
*S* = 1.01	(Δ/σ)_max_ < 0.001
4225 reflections	Δρ_max_ = 0.20 e Å^−^^3^
194 parameters	Δρ_min_ = −0.25 e Å^−^^3^

### Special details

**Table:**

Geometry. All e.s.d.'s (except the e.s.d. in the dihedral angle between two l.s. planes) are estimated using the full covariance matrix. The cell e.s.d.'s are taken into account individually in the estimation of e.s.d.'s in distances, angles and torsion angles; correlations between e.s.d.'s in cell parameters are only used when they are defined by crystal symmetry. An approximate (isotropic) treatment of cell e.s.d.'s is used for estimating e.s.d.'s involving l.s. planes.

### Fractional atomic coordinates and isotropic or equivalent isotropic displacement parameters (Å^2^)

**Table:**

	*x*	*y*	*z*	*U*_iso_*/*U*_eq_	
O1	0.1756 (4)	0.94348 (18)	0.03395 (11)	0.0372 (6)	
C1	0.2580 (7)	0.8418 (3)	0.04912 (17)	0.0368 (9)	
H1	0.3478	0.8095	0.0148	0.044*	
C2	0.4157 (6)	0.8508 (3)	0.10747 (17)	0.0339 (8)	
H2	0.5873	0.8361	0.0990	0.041*	
C3	0.3744 (6)	0.9665 (2)	0.12881 (17)	0.0306 (8)	
H3	0.5155	1.0090	0.1166	0.037*	
C4	0.1536 (6)	1.0022 (2)	0.09017 (16)	0.0309 (8)	
H4	0.0025	0.9832	0.1114	0.037*	
C5	0.1502 (6)	1.1182 (3)	0.07239 (18)	0.0360 (9)	
H5	0.3020	1.1371	0.0514	0.043*	
C6	−0.0661 (6)	1.1468 (3)	0.03179 (19)	0.0392 (9)	
H6A	−0.0244	1.2023	0.0026	0.047*	
H6B	−0.1248	1.0854	0.0093	0.047*	
O7	−0.2414 (4)	1.18276 (17)	0.07540 (12)	0.0391 (6)	
C8	−0.1090 (6)	1.2369 (3)	0.12245 (19)	0.0393 (9)	
O9	0.1197 (4)	1.18174 (19)	0.12615 (13)	0.0470 (7)	
C10	−0.0637 (8)	1.3514 (3)	0.1055 (2)	0.0489 (11)	
H10A	−0.2159	1.3882	0.1027	0.073*	
H10B	0.0184	1.3548	0.0664	0.073*	
H10C	0.0354	1.3840	0.1367	0.073*	
C11	−0.2428 (8)	1.2243 (3)	0.18262 (19)	0.0554 (11)	
H11A	−0.2530	1.1505	0.1930	0.083*	
H11B	−0.4035	1.2530	0.1786	0.083*	
H11C	−0.1572	1.2614	0.2147	0.083*	
O12	0.0607 (5)	0.77711 (19)	0.06819 (12)	0.0397 (6)	
C13	0.1517 (6)	0.7096 (3)	0.11560 (17)	0.0346 (9)	
O14	0.3131 (4)	0.77660 (18)	0.14980 (11)	0.0362 (6)	
C15	0.2887 (8)	0.6163 (3)	0.08712 (19)	0.0489 (11)	
H15A	0.3527	0.5723	0.1194	0.073*	
H15B	0.4198	0.6425	0.0621	0.073*	
H15C	0.1798	0.5756	0.0619	0.073*	
C16	−0.0501 (7)	0.6743 (3)	0.1567 (2)	0.0484 (11)	
H16A	−0.1272	0.7352	0.1748	0.073*	
H16B	0.0138	0.6299	0.1889	0.073*	
H16C	−0.1670	0.6352	0.1330	0.073*	
C3'	0.3314 (6)	0.9814 (3)	0.19700 (17)	0.0339 (8)	
H3'1	0.2859	1.0546	0.2044	0.041*	
H3'2	0.1959	0.9372	0.2095	0.041*	
N4'	0.5468 (6)	0.9550 (2)	0.23638 (16)	0.0373 (7)	
O5'	0.7368 (4)	0.9282 (2)	0.21135 (13)	0.0518 (8)	
O6'	0.5199 (5)	0.9616 (3)	0.29175 (14)	0.0612 (9)	

### Atomic displacement parameters (Å^2^)

**Table:**

	*U*^11^	*U*^22^	*U*^33^	*U*^12^	*U*^13^	*U*^23^
O1	0.0535 (15)	0.0301 (13)	0.0281 (14)	0.0051 (11)	−0.0038 (12)	−0.0004 (12)
C1	0.045 (2)	0.0324 (19)	0.033 (2)	0.0065 (17)	0.0013 (17)	−0.0011 (16)
C2	0.0328 (19)	0.0309 (18)	0.038 (2)	0.0052 (15)	0.0025 (17)	0.0038 (17)
C3	0.0258 (17)	0.0283 (19)	0.038 (2)	−0.0020 (13)	−0.0002 (15)	−0.0018 (16)
C4	0.0273 (17)	0.0285 (18)	0.037 (2)	−0.0028 (14)	−0.0016 (16)	−0.0007 (17)
C5	0.0299 (18)	0.0302 (19)	0.048 (2)	0.0000 (14)	−0.0025 (18)	0.0015 (18)
C6	0.038 (2)	0.0311 (19)	0.048 (2)	0.0058 (16)	−0.0062 (18)	0.0003 (19)
O7	0.0308 (13)	0.0349 (13)	0.0517 (17)	−0.0016 (11)	−0.0035 (12)	−0.0014 (13)
C8	0.038 (2)	0.0284 (19)	0.052 (3)	0.0024 (15)	−0.0039 (18)	−0.0024 (18)
O9	0.0418 (15)	0.0345 (14)	0.0646 (19)	0.0089 (11)	−0.0173 (13)	−0.0132 (14)
C10	0.055 (2)	0.030 (2)	0.062 (3)	−0.0002 (18)	0.003 (2)	0.003 (2)
C11	0.068 (3)	0.046 (2)	0.052 (3)	−0.004 (2)	0.002 (2)	0.003 (2)
O12	0.0432 (14)	0.0323 (13)	0.0436 (15)	−0.0020 (11)	−0.0114 (12)	0.0004 (13)
C13	0.040 (2)	0.0257 (18)	0.038 (2)	−0.0024 (15)	−0.0072 (17)	−0.0009 (16)
O14	0.0408 (14)	0.0327 (13)	0.0352 (15)	−0.0045 (11)	−0.0058 (12)	0.0045 (12)
C15	0.065 (3)	0.0281 (19)	0.053 (3)	0.0049 (18)	−0.004 (2)	−0.0008 (19)
C16	0.045 (2)	0.042 (2)	0.059 (3)	−0.0028 (18)	0.000 (2)	0.006 (2)
C3'	0.0245 (17)	0.038 (2)	0.040 (2)	−0.0017 (15)	−0.0016 (16)	−0.0010 (17)
N4'	0.0387 (18)	0.0316 (17)	0.042 (2)	−0.0026 (14)	−0.0078 (16)	0.0001 (16)
O5'	0.0312 (14)	0.0693 (19)	0.0548 (19)	0.0061 (14)	−0.0057 (14)	0.0012 (15)
O6'	0.0627 (19)	0.085 (2)	0.0355 (18)	0.0003 (16)	−0.0099 (16)	−0.0045 (17)

### Geometric parameters (Å, º)

**Table:**

O1—C1	1.399 (4)	C8—C10	1.511 (5)
O1—C4	1.429 (4)	C10—H10A	0.9600
C1—O12	1.420 (4)	C10—H10B	0.9600
C1—C2	1.537 (5)	C10—H10C	0.9600
C1—H1	0.9800	C11—H11A	0.9600
C2—O14	1.427 (4)	C11—H11B	0.9600
C2—C3	1.547 (5)	C11—H11C	0.9600
C2—H2	0.9800	O12—C13	1.424 (4)
C3—C3'	1.506 (5)	C13—O14	1.432 (4)
C3—C4	1.542 (5)	C13—C16	1.491 (5)
C3—H3	0.9800	C13—C15	1.527 (5)
C4—C5	1.514 (4)	C15—H15A	0.9600
C4—H4	0.9800	C15—H15B	0.9600
C5—O9	1.422 (4)	C15—H15C	0.9600
C5—C6	1.523 (5)	C16—H16A	0.9600
C5—H5	0.9800	C16—H16B	0.9600
C6—O7	1.424 (4)	C16—H16C	0.9600
C6—H6A	0.9700	C3'—N4'	1.498 (4)
C6—H6B	0.9700	C3'—H3'1	0.9700
O7—C8	1.426 (4)	C3'—H3'2	0.9700
C8—O9	1.441 (4)	N4'—O6'	1.210 (4)
C8—C11	1.504 (6)	N4'—O5'	1.225 (4)
			
C1—O1—C4	107.6 (2)	C11—C8—C10	113.0 (3)
O1—C1—O12	110.3 (3)	C5—O9—C8	109.2 (3)
O1—C1—C2	107.9 (3)	C8—C10—H10A	109.5
O12—C1—C2	103.7 (3)	C8—C10—H10B	109.5
O1—C1—H1	111.5	H10A—C10—H10B	109.5
O12—C1—H1	111.5	C8—C10—H10C	109.5
C2—C1—H1	111.5	H10A—C10—H10C	109.5
O14—C2—C1	104.8 (3)	H10B—C10—H10C	109.5
O14—C2—C3	111.7 (3)	C8—C11—H11A	109.5
C1—C2—C3	103.4 (3)	C8—C11—H11B	109.5
O14—C2—H2	112.1	H11A—C11—H11B	109.5
C1—C2—H2	112.1	C8—C11—H11C	109.5
C3—C2—H2	112.1	H11A—C11—H11C	109.5
C3'—C3—C4	111.8 (3)	H11B—C11—H11C	109.5
C3'—C3—C2	115.7 (3)	C1—O12—C13	106.5 (3)
C4—C3—C2	103.3 (3)	O12—C13—O14	103.8 (2)
C3'—C3—H3	108.6	O12—C13—C16	110.2 (3)
C4—C3—H3	108.6	O14—C13—C16	109.3 (3)
C2—C3—H3	108.6	O12—C13—C15	110.1 (3)
O1—C4—C5	106.6 (3)	O14—C13—C15	110.9 (3)
O1—C4—C3	104.1 (3)	C16—C13—C15	112.2 (3)
C5—C4—C3	115.5 (3)	C2—O14—C13	107.5 (2)
O1—C4—H4	110.1	C13—C15—H15A	109.5
C5—C4—H4	110.1	C13—C15—H15B	109.5
C3—C4—H4	110.1	H15A—C15—H15B	109.5
O9—C5—C4	109.8 (3)	C13—C15—H15C	109.5
O9—C5—C6	104.2 (3)	H15A—C15—H15C	109.5
C4—C5—C6	112.7 (3)	H15B—C15—H15C	109.5
O9—C5—H5	110.0	C13—C16—H16A	109.5
C4—C5—H5	110.0	C13—C16—H16B	109.5
C6—C5—H5	110.0	H16A—C16—H16B	109.5
O7—C6—C5	102.9 (3)	C13—C16—H16C	109.5
O7—C6—H6A	111.2	H16A—C16—H16C	109.5
C5—C6—H6A	111.2	H16B—C16—H16C	109.5
O7—C6—H6B	111.2	N4'—C3'—C3	113.9 (3)
C5—C6—H6B	111.2	N4'—C3'—H3'1	108.8
H6A—C6—H6B	109.1	C3—C3'—H3'1	108.8
C6—O7—C8	106.2 (2)	N4'—C3'—H3'2	108.8
O7—C8—O9	104.8 (3)	C3—C3'—H3'2	108.8
O7—C8—C11	108.5 (3)	H3'1—C3'—H3'2	107.7
O9—C8—C11	109.2 (3)	O6'—N4'—O5'	124.1 (3)
O7—C8—C10	111.7 (3)	O6'—N4'—C3'	116.8 (3)
O9—C8—C10	109.3 (3)	O5'—N4'—C3'	119.1 (3)
			
C4—O1—C1—O12	82.3 (3)	C6—O7—C8—O9	−32.8 (3)
C4—O1—C1—C2	−30.3 (3)	C6—O7—C8—C11	−149.3 (3)
O1—C1—C2—O14	126.3 (3)	C6—O7—C8—C10	85.4 (3)
O12—C1—C2—O14	9.3 (3)	C4—C5—O9—C8	−115.4 (3)
O1—C1—C2—C3	9.3 (4)	C6—C5—O9—C8	5.6 (4)
O12—C1—C2—C3	−107.7 (3)	O7—C8—O9—C5	16.0 (4)
O14—C2—C3—C3'	23.3 (4)	C11—C8—O9—C5	132.1 (3)
C1—C2—C3—C3'	135.5 (3)	C10—C8—O9—C5	−103.8 (3)
O14—C2—C3—C4	−99.1 (3)	O1—C1—O12—C13	−144.5 (3)
C1—C2—C3—C4	13.1 (3)	C2—C1—O12—C13	−29.2 (3)
C1—O1—C4—C5	161.0 (3)	C1—O12—C13—O14	38.3 (3)
C1—O1—C4—C3	38.5 (3)	C1—O12—C13—C16	155.2 (3)
C3'—C3—C4—O1	−155.8 (3)	C1—O12—C13—C15	−80.4 (3)
C2—C3—C4—O1	−30.8 (3)	C1—C2—O14—C13	13.7 (3)
C3'—C3—C4—C5	87.7 (4)	C3—C2—O14—C13	125.0 (3)
C2—C3—C4—C5	−147.3 (3)	O12—C13—O14—C2	−31.8 (3)
O1—C4—C5—O9	177.9 (3)	C16—C13—O14—C2	−149.4 (3)
C3—C4—C5—O9	−67.0 (4)	C15—C13—O14—C2	86.4 (3)
O1—C4—C5—C6	62.2 (4)	C4—C3—C3'—N4'	−176.0 (3)
C3—C4—C5—C6	177.3 (3)	C2—C3—C3'—N4'	66.2 (4)
O9—C5—C6—O7	−25.0 (3)	C3—C3'—N4'—O6'	−177.3 (3)
C4—C5—C6—O7	94.0 (3)	C3—C3'—N4'—O5'	2.6 (4)
C5—C6—O7—C8	35.7 (3)		

### Hydrogen-bond geometry (Å, º)

**Table:**

*D*—H···*A*	*D*—H	H···*A*	*D*···*A*	*D*—H···*A*
C3′—H3′2···O5′^i^	0.97	2.53	3.355 (4)	143
C1—H1···O12^ii^	0.98	2.41	3.386 (5)	178
C15—H15*A*···O6′^iii^	0.96	2.48	3.433 (5)	174

Symmetry codes: (i) *x*−1, *y*, *z*; (ii) *x*+1/2, −*y*+3/2, −*z*; (iii) −*x*+1, *y*−1/2, −*z*+1/2.

## References

[bb1] Albrecht, H. P. & Moffatt, J. G. (1970). *Tetrahedron Lett.* **11**, 1063–1066. 10.1016/s0040-4039(01)97908-0 5439237

[bb2] Altona, C. & Sundaralingam, M. (1972). *J. Am. Chem. Soc.* **94**, 8205–8212. 10.1021/ja00778a043 5079964

[bb3] Burla, M. C., Caliandro, R., Camalli, M., Carrozzini, B., Cascarano, G. L., Giacovazzo, C., Mallamo, M., Mazzone, A., Polidori, G. & Spagna, R. (2012). *J. Appl. Cryst.* **45**, 357–361.

[bb4] Farrugia, L. J. (2012). *J. Appl. Cryst.* **45**, 849–854.

[bb5] Filichev, V. V., Brandt, M. & Pedersen, E. B. (2001). *Carbohydr. Res.* **333**, 115–122. 10.1016/s0008-6215(01)00132-x 11448671

[bb6] Filichev, V. V. & Pedersen, E. B. (2001). *Tetrahedron*, **57**, 9163–9168.

[bb7] Grigorjeva, J., Uzuleņa, J., Rjabovs, V. & Turks, M. (2015). *Chem. Heterocycl. Compd.* **51**, 883–890.

[bb8] Groom, C. R. & Allen, F. H. (2014). *Angew. Chem. Int. Ed.* **53**, 662–671. 10.1002/anie.201306438 24382699

[bb9] Lugiņina, J., Rjabovs, V., Belyakov, S. & Turks, M. (2012). *Carbohydr. Res.* **350**, 86–89. 10.1016/j.carres.2011.12.020 22281179

[bb10] Lugiņina, J., Rjabovs, V., Belyakov, S. & Turks, M. (2013). *Tetrahedron Lett.* **54**, 5328–5331.

[bb11] Mackeviča, J., Ostrovskis, P., Leffler, H., Nilsson, U. J., Rudovica, V., Viksna, A., Belyakov, S. & Turks, M. (2014). *ARKIVOC*, (**iii**), 90–112.

[bb12] Nonius (1997). *KappaCCD Server Software*. Nonius BV, Delft, The Netherlands.

[bb13] Otwinowski, Z. & Minor, W. (1997). *Methods in Enzymology*, Vol. 276, *Macromolecular Crystallography*, Part A, edited by C. W. Carter Jr & R. M. Sweet, pp. 307–326. New York: Academic Press.

[bb14] Rjabova, J., Rjabovs, V., Moreno Vargas, A. J., Clavijo, E. M. & Turks, M. (2012). *Cent. Eur. J. Chem.* **10**, 386–394.

[bb27] Rjabovs, V., Mishnev, A., Kiselovs, G. & Turks, M. (2014). *Acta Cryst.* E**70**, o524–o525. 10.1107/S1600536814007387 PMC4011227 24860338

[bb15] Rjabovs, V., Ostrovskis, P., Posevins, D., Kiselovs, G., Kumpiņš, V., Mishnev, A. & Turks, M. (2015*a*). *Eur. J. Org. Chem.* pp. 5572–5584.

[bb26] Rjabovs, V., Stepanovs, D. & Turks, M. (2015*b*). *Acta Cryst.* E**71**, 1212–1215. 10.1107/S2056989015017582 PMC4647424 26594409

[bb16] Rjabovs, V. & Turks, M. (2013). *Tetrahedron*, **69**, 10693–10710.

[bb17] Rozners, E., Katkevica, D., Bizdena, E. & Strömberg, R. (2003). *J. Am. Chem. Soc.* **125**, 12125–12136. 10.1021/ja0360900 14518999

[bb18] Sheldrick, G. M. (2015). *Acta Cryst.* C**71**, 3–8.

[bb19] Spek, A. L. (2009). *Acta Cryst.* D**65**, 148–155. 10.1107/S090744490804362X PMC2631630 19171970

[bb20] Taha, H. A., Richards, M. R. & Lowary, T. L. (2013). *Chem. Rev.* **113**, 1851–1876. 10.1021/cr300249c 23072490

[bb21] Turks, M., Rodins, V., Rolava, E., Ostrovskis, P. & Belyakov, S. (2013). *Carbohydr. Res.* **375**, 5–15. 10.1016/j.carres.2013.04.008 23665157

[bb22] Turks, M., Vēze, K., Kiselovs, G., Mackeviča, J., Lugiņina, J., Mishnev, A. & Marković, D. (2014). *Carbohydr. Res.* **391**, 82–88. 10.1016/j.carres.2014.03.003 24785391

[bb23] Uzuleņa, J., Rjabovs, V., Moreno-Vargas, A. J. & Turks, M. (2015). *Chem. Heterocycl. Compd*, **51**, 664–671.

[bb24] Westrip, S. P. (2010). *J. Appl. Cryst.* **43**, 920–925.

[bb25] Zhang, Q., Ke, Y., Cheng, W., Li, P. & Liu, H. (2011). *Acta Cryst.* E**67**, o1402. 10.1107/S1600536811017314 PMC3120445 21754787

